# Biomechanical evaluation of an allograft fixation system for ACL reconstruction

**DOI:** 10.3389/fbioe.2022.1000624

**Published:** 2022-11-03

**Authors:** Emir Benca, Kenneth P. van Knegsel, Ivan Zderic, Jan Caspar, Andreas Strassl, Lena Hirtler, Christoph Fuchssteiner, Boyko Gueorguiev, Reinhard Windhager, Harald Widhalm, Peter Varga

**Affiliations:** ^1^ Department of Orthopedics and Trauma Surgery, Medical University of Vienna, Vienna, Austria; ^2^ AO Research Institute Davos, Davos Platz, Switzerland; ^3^ Department of Orthopedics and Trauma Surgery, Cantonal Hospital of Lucerne, Lucerne, Switzerland; ^4^ Department of Biomedical Imaging and Image-Guided Therapy, Medical University of Vienna, Vienna, Austria; ^5^ Division of Anatomy, Center for Anatomy and Cell Biology, Medical University of Vienna, Vienna, Austria

**Keywords:** ACL, fixation, human specimens, allograft, *in vitro*, biomechanical study

## Abstract

The purpose of this study was to compare the biomechanical stability, especially graft slippage of an allograft screw and a conventional interference screw for tibial implant fixation in ACL reconstruction. Twenty-four paired human proximal tibia specimens underwent ACL reconstruction, with the graft in one specimen of each pair fixed using the allograft screw and the other using the conventional interference screw. Specimens were subjected to cyclic tensile loading until failure. The two fixation methods did not show any statistical difference in load at graft slippage (*p* = 0.241) or estimated mean survival until slippage onset (*p* = 0.061). The ultimate load and the estimated mean survival until failure were higher for the interference screw (*p* = 0.04, and *p* = 0.018, respectively). Graft displacement at ultimate load reached values of up to 7.2 (interference screw) and 11.3 mm (allograft screw). The allograft screw for implant fixation in ACL reconstruction demonstrated comparable behavior in terms of graft slippage to the interference screw but underperformed in terms of ultimate load. However, the ultimate load, occurring at progressive graft slippage, may not be considered a direct indicator of clinical failure.

## 1 Introduction

Bioabsorbable and metal interference screws have been used for implant fixation in the anterior cruciate ligament (ACL) reconstruction with good midterm results (Lajtai et al., 2001; Pinczewski et al., 2007). Bioabsorbable screw materials include biodegradable polymers such as poly-glycolide acid (PGA), poly-L-lactic acid (PLLA), and poly-D, L-lactic acid. Depending on the polymer composition, they express different material characteristics. Highly crystalline PLLA and poly(lactic-co-glycolic acid) (PLGA) stereocopolymers with a low D,L amount have mechanical advantages, such as higher stiffness or lower viscoplastic deformation (Schlichting et al., 2006), while their degradation process is slow lasting up to several years and is often incomplete due to possible accumulation of insoluble crystalline implant remnants. A 2-year follow-up study found that only 5% PLLA screws have resorbed (Warden et al., 1999). Amorphous poly-(L-co-D,L-lactide) stereocopolymers with a high D,L amount and the porous poly-(D,L-lactide) degrade completely within one to 2 years, but have low initial fixation strength (Brand J. et al., 2000). Finally, in a recent study (median follow-up: 32 months) with 925 ACL reconstruction procedures using bioabsorbable screws in 858 patients, aged less than 18 years, 9.6% developed a screw-related problem, most commonly screw site pain and screw prominence. The incidence of screw-related complications was reported to be higher in low-volume surgeons compared to high-volume surgeons (16.7% versus 8.7%, respectively). Nevertheless, while surgical skill level improved the clinical outcome, a significant complication incidence remained, related to screw material (Kramer et al., 2020). On the other hand, metal screws provide higher fixation stability but they have been reported to damage the tendon grafts by their sharp threads (Giurea et al., 1999). Furthermore, Magnetic Resonance Imaging (MRI) is difficult due to presence of artefacts caused by metal screws. Finally, surgical revision might be challenging when bone ingrowth has progressed. Unlike softer materials, metal screws cannot be drilled through in revision surgery. A hardware-free alternative to interference screws is the use of bone-tendon-bone autografts with good long-term results. However, this technique is opposed by a certain degree of technical difficulty and limited applicability in patients with poor bone quality (Hertel et al., 2005).

The recently introduced commercially available allograft osteosynthesis system, the Shark Screw (surgebright GmbH, Lichtenberg bei Linz, Austria), has already been applied in foot, and hand surgery (Pastl and Schimetta, 2021). Shark Screw is a threaded cylinder milled from human cortical bone and sterilized using peracetic acid/ethanol. The advantages compared to bioabsorbable and/or metal implants are the ingrowth of the surrounding bone tissue in the implant, and the elimination of potential foreign body reaction and of obsolete device removal. A case study reported that 10 weeks post-operatively the Shark Screw allograft used for first metatarsophalangeal joint arthrodesis was vascularized by the ingrowth of vessels into the majority of Haversian pore network, and bone remodeling was in progress including osteoclastic and osteoblastic activity. This resulted in creation of new host bone at the bone-graft interface without signs of immunological rejection (Brcic et al., 2021). Another case series with 32 patients with an average follow-up time of 1 year reported very good clinical results, including high patient satisfaction, low postoperative pain level and no implant failure or loosening (Pastl and Schimetta, 2021). Furthermore, there are no potential imaging artefacts as experienced from metal devices. A novel system, specially designed for fixation of ACL grafts–Shark Screw ACL–is available in 8.0 × 21 mm size. Given the first promising clinical results in osteosynthesis in several other anatomical sites, the use of Shark Screw ACL for management of ACL graft fixation is conceivable. However, prior to clinical application, the biomechanical performance of the fixation using Shark Screw ACL has to be evaluated.

Numerous aspects must be considered for physiologically relevant biomechanical testing of ACL fixations, but not all have been carefully addressed in previous studies. Most investigations on this topic have evaluated biomechanical properties in terms of stiffness and ultimate load of ACL graft fixation methods in animal bone, predominantly porcine. However, two studies (Magen et al., 1999; Bailey et al., 2004) demonstrated that the use of porcine bone significantly overestimates the yield and ultimate loads for fixation using an interference screw, compared to young human bone. The mean ultimate and yield load in those studies were higher by 87% and 122%, respectively, for the interference screw inserted in animal tissue compared to young human bone. Both studies concluded that the structural properties of a fixation method may not be the same in animal and human tissue. Following an ACL reconstruction, the tibial side may be biomechanically less competent for several reasons. The bone density in femoral- and tibial metaphysis exhibits a significant difference with greater values in the femur (Brand J. C. et al., 2000), implying higher stability of the femoral fixation and rendering the tibial implant to be the weak point, as confirmed in several previous studies (Mayr et al., 2015; Smith et al., 2018). Furthermore, the forces applied to the ACL are oriented parallel to the tibial graft tunnel (Malek et al., 1996; Brand J. et al., 2000), which will further facilitate mechanical failure.

Finally, the interference screw compresses the graft against the bone in a press-fit manner. Therefore, the strength of the fixation depends on local bone quality and insertion torque, as it was shown for a single biodegradable interference screw –BioScrew (Linvatec, Largo, FL, United States) in an *in vitro* study (Brand J. C. et al., 2000). A relation between the bone mineral density (BMD) and fixation strength would allow for preoperative fixation strength estimation. Similarly, a possible correlation between the insertion torque and the fixation strength would allow surgeons to use the latter as an intraoperative predictor for final strength. Nevertheless, these relations have not been verified in further studies.

Therefore, the aim of this study was to compare the biomechanical stability, especially the onset of slippage of the Shark Screw ACL for tibial implant fixation in ACL reconstruction compared to a commonly used bioabsorbable interference screw in human anatomic specimens. The authors hypothesise that the two fixation devices will demonstrate no significant differences in terms of fixation strength.

## 2 Materials and methods

### 2.1 Specimens

Twenty-four paired anatomic specimens of human proximal tibiae with preserved semitendinosus and gracilis tendons were obtained for this study from three female and nine male donors aged 72.7 ± 5.6 years (mean ± standard deviation, SD, range 62–79 years) at the Medical University of Vienna (Vienna, Austria). The donors had given their written consent during lifetime for their body to be used for research and education. The study was approved by the Ethics Committee of the Medical University of Vienna (2144/2020). The donor age was limited between 18 and 80 years. Furthermore, all specimens were inspected for bone damage, tendon degeneration or previous surgical treatment. The specimens were thawed at room temperature for 24 h, kept moist with 0.9% saline solution during the preparation and tested at room temperature. The proximal part of each tibia was cut at a length of 110 mm using an oscillating saw and carefully stripped of soft tissues. Following the surgical procedure (described below), the distal 30 mm of each specimen was embedded in polymethylmethacrylate (PMMA, SCS-Beracryl D28, Suter Kunststoffe AG, Fraubrunnen, Switzerland). Projected line laser beams ensured that both the graft tunnel and the graft itself were in line with the test system axis, i.e. in the planned loading direction, representing the mechanical worst-case scenario for pullout failure.

### 2.2 QCT scanning

BMD was assessed from quantitative computed tomography (QCT) scans for correlation with the mechanical parameters, aiming to account for the high donor age and the associated potentially lower bone quality. A 128-slice multi-detector computed tomography (MDCT) scanner (Siemens SOMATOM Edge Plus, Siemens Healthineers, Forchheim, Germany) was used with the following settings: 120 kV tube voltage, 90 mAs tube current-time-product, 128 × 0.6 mm collimation, 1 s rotation time and 0.8 pitch. The specimens were placed pairwise in longitudinal direction on the scanner bed together with a calibration phantom (BMD Referencebody, Siemens, München, Germany). Images were reconstructed using the following parameters: 512 × 512 matrix size, 1 mm slice thickness, 0.8 mm slice increment and Br60 reconstruction kernel. The reconstructed field-of-view (FOV) was limited to the size of a single specimen.

The BMD assessment was performed using Mimics 22 (Materialise NV, Leuven, Belgium). After importing the QCT image series, a Hounsfield units (HU) histogram was created for the most proximal 100 mm of each specimen and exported using the 3D Histogram function. Bone tissue was assumed in the range of 226–3071 HU, as defined in Mimics using global thresholds segmentation function for bone. Volumetric BMD (mg/cm³) was then converted from the HU by multiplying with a calibration factor. The latter was determined as the mean HU value of the bone compartment of the reference body, divided by 200 mg Calcium Hydroxyapatite per ml, normalized with a unique scaling factor. The BMD range was limited between 100 and 1,400 mg CaHA/cm³ to restrict the effect of residual air and to minimize artefacts.

### 2.3 Surgical procedure

**FIGURE 1:**
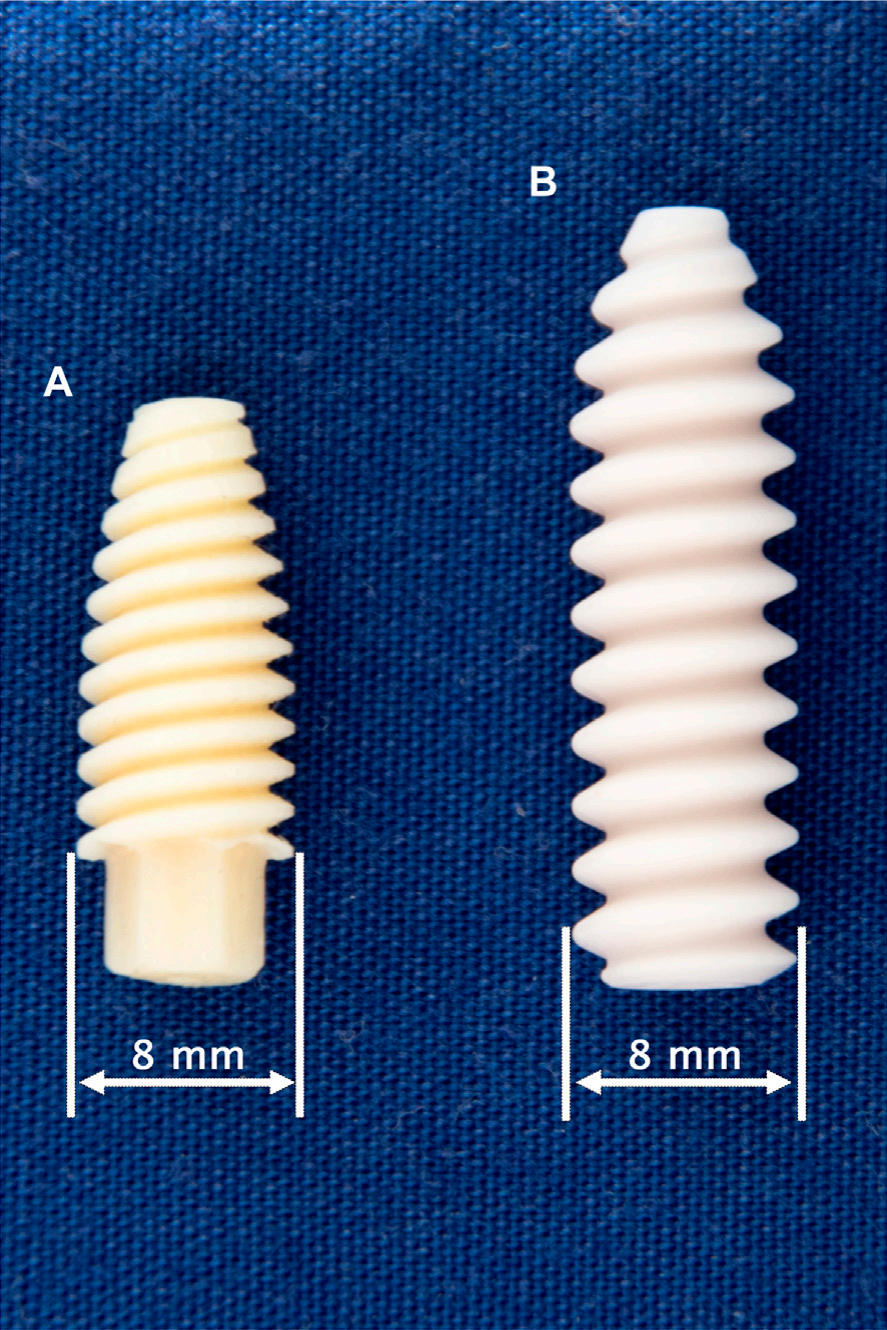
Tested fixation screws: 8 × 21 mm Shark Screw ACL (surgebright GmbH, Lichtenberg bei Linz, Austria) **(A)** and 8 × 28 mm BioComposite Interference Screw (Arthrex Inc., Naples, FL, United States) **(B)**.

The semitendinosus and gracilis tendons were harvested in their full length, quadrupled and, when necessary, longitudinally trimmed to match an 8 mm sizing sleeve. The distal 30–40 mm of the graft were then sutured in a running whipstitch manner using two No. 1 Polysorb sutures (Covidien Ilc, Mansfield, MA, United States) and pretensioned at 50 N, based on previous pilot tests using animal tissue. A guidewire was placed anatomically in lateral to medial direction through the origin of the original ACL with an angle of 65°, followed by incremental drilling of the tibial tunnel with a 5 mm and an 8 mm diameter drill. The graft was inserted into the distal tunnel end and subsequently pulled into proximal direction. In the interference screw group, a 1.0 mm guidewire was placed laterally in the interface between the tendon graft and the tunnel, followed by placement of a fully threaded 8 × 28 mm BioComposite Interference Screw (Arthrex Inc., Naples, FL, United States) (Figure 1B) over the same guidewire. The screwdriver was mounted onto a digital torque screwdriver (DTS-101; Sushma Industries, Bangalore, Karnataka, India) with a capacity of 10 Nm, used to measure the maximum insertion torque in all specimens. The final position of the screws was controlled via x-ray imaging.

The insertion of the Shark Screw ACL (Figure 1A) was performed in a similar manner to the insertion of the interference screws with three minor modifications as follows. First, the proximal tunnel entrance was overdrilled with a 9 mm drill at a depth of 2–3 mm to prevent the contact of cortical bone with the Shark Screw ACL and the resulting possible implant damage. Second, based on the surgical protocol, the screw was guided over either a guidewire or a strong strained suture. Accordingly, either a 1.0 mm guidewire or a FiberWire #2 (Arthrex Inc., Naples, FL, United States) was used. Third, the use of the rather long and heavy digital torque screwdriver was not feasible in the Shark Screw ACL group as it generated bending moments causing screw breakage. Thus, the insertion torque was assessed in only five specimens of this group and no measurements were performed for the remaining ones. The targeted final position of the screws – controlled via x-ray imaging – was identical in both groups.

### 2.4 Biomechanical testing

**FIGURE 2:**
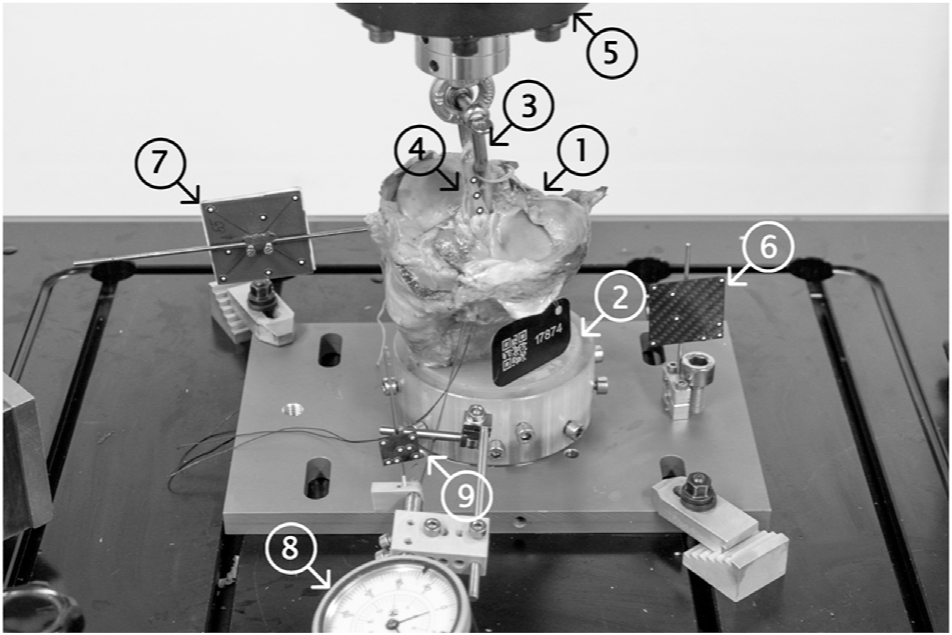
Test setup showing a mounted specimen ready for biomechanical testing. The proximal 110 mm of the human tibia (1) were distally embedded using PMMA (2) and fixed into the load frame. A 5.5 mm D-shackle (3) was inserted and secured into the loop of the graft (4) and the load actuator (5). Single components (load frame (6), specimen (7) and tendon graft (4)) were marked using retro-reflective markers and their displacements were tracked using an optical motion tracking system. A dial gauge (8), rigidly fixed to the load frame, was used to pretension the suture at the distal end of the tendon graft (9) and tracked to monitor the identical migration of the tendon in the tunnel (slippage) otherwise not visible to the tracking system.

The embedded specimens were mounted in an electrodynamic test system (Acumen; MTS Systems Corporation, Eden Prairie, MN, United States) (Figure 2). A tensile load was applied along the graft tunnel axis, mimicking the worst-case scenario and approximating physiological loading direction in the tibia for both knee flexion and extension. The specimens were preloaded at 50 N for 10 s to minimize play between single components. This value was chosen based on pilot tests using animal bone and graft tissue ensuring elimination of any relative motions between various components without compromising the integrity of the specimens. Afterwards the specimens were loaded cyclically at a rate of 1 Hz with a constant valley load of 50 N and a peak load level monotonically increased at a rate of 0.1 N/cycle, starting from 50 N until the catastrophic construct failure. Catastrophic construct failure was potentially defined as tendon and/or implant pullout, tendon rupture or bone breakage causing a clear drop of the load-displacement curve. The application of progressively increasing cyclic loading allowed to achieve construct failure of the fixations in specimens with different bone quality within a predefined number of cycles. Load and displacement data was collected at a rate of 128 Hz. Relative movements of the tendon, bone, and suture at the distal end of the tibial tunnel were measured via monitoring of markers with a stereographic optical motion tracking system (Aramis SRX; GOM GmbH, Braunschweig, Germany) at a rate of up to 115 Hz. The tracking of the suture at the distal end of the graft (Benca et al., 2021) enabled the monitoring of the graft slippage. Slippage onset was defined as a displacement of the distal graft end bigger than 0.1 mm. The value of 0.1 mm was chosen to exceed the lowest detectable displacement limit that can clearly be distinguished from signal noise.

It has to be noted that some data of fourteen specimens used in this study has been previously reported in a paper concerning general aspects in measurement of structural characteristics and mechanical behavior of ACL implant fixations (Benca et al., 2021).

### 2.5 Statistical analysis

Outcome parameters of interest were tested for normality of distribution within each group using the Shapiro-Wilk test. Independent-Samples t-test and Wilcoxon Signed-Rank test were performed to screen for significant differences between the two fixation groups in terms of BMD, insertion torque, load at slippage onset, number of cycles at slippage onset, ultimate load, and number of cycles at slippage onset. The relation of construct failure to loading cycles was assessed by use of Kaplan-Meier survival analysis and Mantel-Cox test for screening of differences between the implant groups. The Pearson product-moment correlation coefficient was computed to investigate linear correlations between 1) BMD of the two groups, 2) BMD and insertion torque, 3) BMD and load at slippage, 4) BMD and ultimate load, 5) load at slippage onset and ultimate load, and 6) insertion torque and load at slippage onset. To investigate if the inter-individual characteristics have an effect on the outcome variables, further correlation analyses between the specimen pairs were performed for 7) load at slippage onset, 8) number of cycles at slippage onset, 9) ultimate load, and 10) number of cycles at slippage onset. Level of significance was set at 0.05 for all statistical tests. The analysis was performed using IBM SPSS Statistics 26 (IBM Corp., Armonk, New York, United States). Descriptive values were calculated in terms of either mean and standard deviation (SD, in case of normal data distribution), or median and interquartile range (IQR, in case of non-normal data distribution).

## 3 Results

**FIGURE 3:**
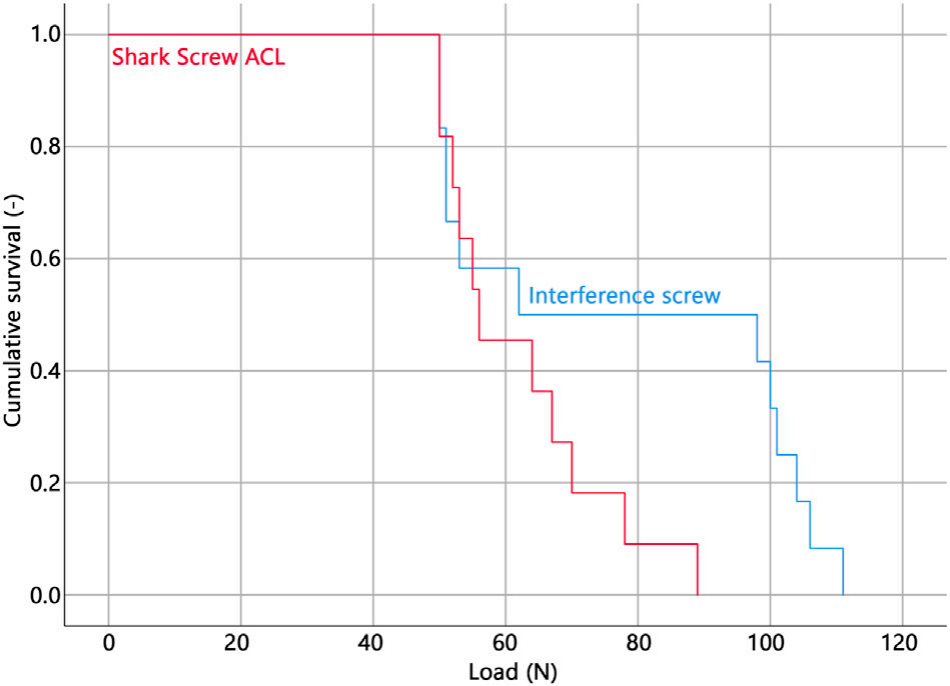
Cumulative survival analysis based on the loads at slippage onset.

**FIGURE 4:**
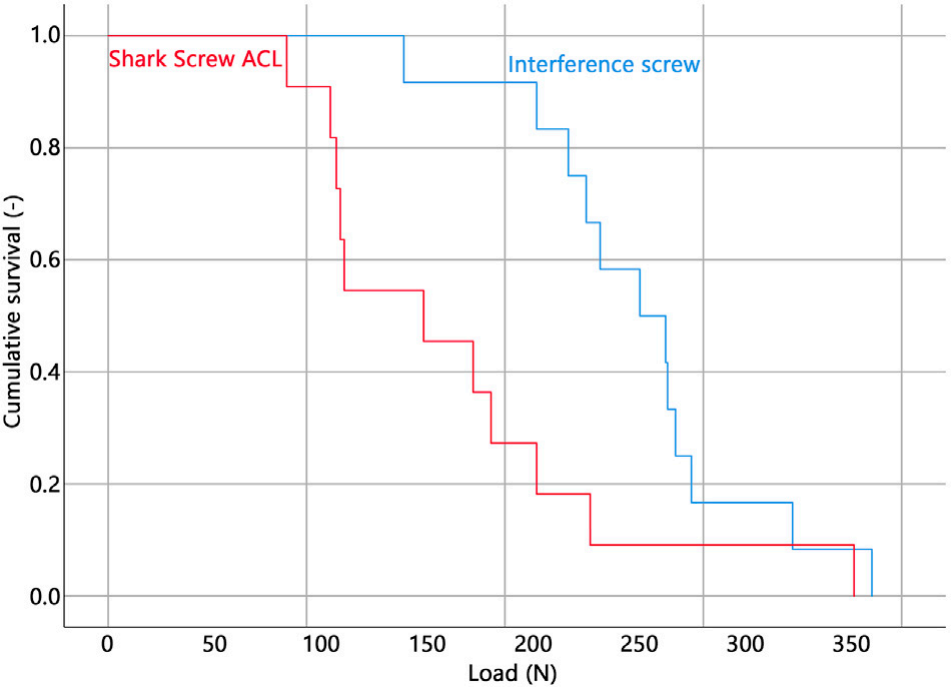
Cumulative survival analysis based on the ultimate loads.

All but one specimen were successfully instrumented and tested. In a single specimen within the Shark Screw ACL group that was instrumented first, the screw head broke during insertion and therefore the specimen was excluded from data analysis. The corresponding contralateral specimen was excluded in the pairwise comparisons. Descriptive results of all investigated parameters of interest are presented together with the outcomes of the corresponding pairwise comparisons in Table 1. BMD, insertion torque, load at slippage onset, and estimated mean survival based on loads at slippage onset were not significantly different between the two groups. (Figure 3). The ultimate load was significantly higher in the interference screw group (*p* = 0.004), which was also the case for the cumulative survival based on the ultimate loads (*p* = 0.018) (Figure 4).

**TABLE 1:** Results of biomechanical tests.

	Interference screw	Shark Screw ACL	Stat. Sign. [95% CI]
BMD (mg/cm³)	829.6 ± 45.6 (750.7–886.8)	830.72 ± 44.07 (768.4–881.8)	0.866 [-15.2, 13.0]
Insertion torque (Nm)	0.817 ± 0.226 (0.45–1.13)	0.822 ± 0.289 (0.44–1.19)^a^	0.556 [-0.348, 0.558]
Load at slippage onset (N)	64.5 (IQR: 51.0–102.5)	56.0 (IQR: 52.0–70.0)	0.241
Cycles until slippage onset (-)	274 (IQR: 12–539)	88 (IQR: 25–407)	0.248
Estimated mean survival until slippage onset (N)	78.0 (63.0–93.2)	62.2 (54.6–96.7)	0.061
Ultimate load (N)	277.3 ± 64.1 (148.7–384.5)	174.9 ± 82.9 (89.8–375.8)	0.04 [40.7, 164.1]
Cycles at ultimate load (-)	2194 ± 555 (981–2942)	1189 ± 858 (394–3260)	0.003 [415, 1594]
Estimated mean survival until ultimate load (N)	268.9 [95% CI: 234.7–303.1]	174.9 [95% CI: 125.9–223.9]	0.018
Displacement at ultimate load (mm)	2.9 (IQR: 2.4–4.9)	2.5 (IQR: 1.3–3.0)	0.182

^a^ Data based on five specimens.

The Kaplan-Meier survivorship results for the two implant groups revealed no significant difference in estimated mean survival until slippage onset. The interference screws (estimated mean survival: 268.9 N) were significantly more resistant to cyclic loading until catastrophic construct failure than the Shark Screw ACL (estimated mean survival: 174.9 N) (*p* = 0.021).

**FIGURE 5:**
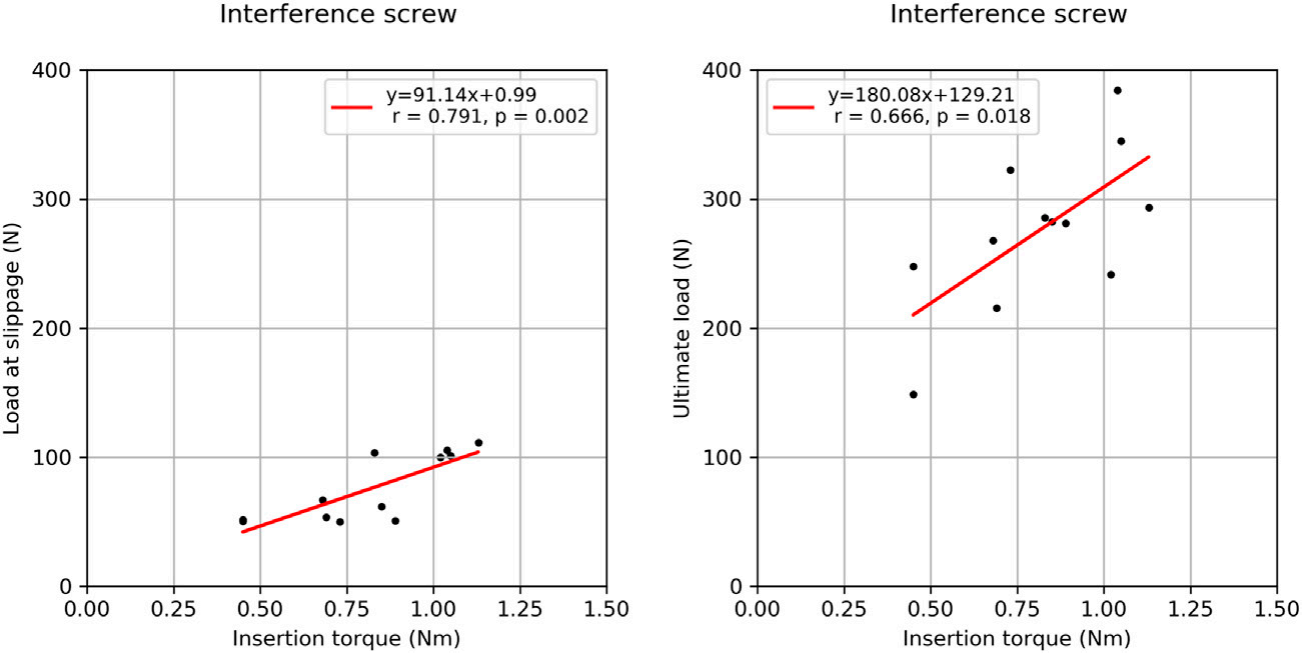
Correlation plots between the insertion torque and load at slippage (left) onset and the insertion torque and ultimate load (right) for the interference screw group.

There was a strong correlation between the BMD of the two groups (*R* ^2^ = 0.796, *p* < 0.001) suggesting similar material properties in paired specimens. There was no significant correlation between BMD and insertion torque, BMD and load at slippage onset, BMD and ultimate load, and ultimate load and load at slippage onset (0.040 < *R* ^2^ < 0.346, 0.057 < p < 0.533). In the interference screw group, insertion torque correlated significantly with load at slippage onset (*R* ^2^ = 0.635, *p* = 0.002) and non-significantly with ultimate load (*R* ^2^ = 0.444, *p* = 0.18) (Figure 5). In the Shark Screw ACL group non-significant correlations were observed between insertion torque and load at slippage onset (*R* ^2^ = 0.759, *p* = 0.129), and between insertion torque and ultimate load (*R* ^2^ = 0.719, *p* = 0.152). There was no significant correlation between the two groups with regard to the load at slippage onset, number of cycles at slippage onset, ultimate load, and the number of cycles at slippage onset (0.057 < *R* ^2^ < 0.480).

The ACL graft was pulled out together with the screw in one specimen from the Shark Screw ACL group. This specimen reached the highest ultimate load (375.8 N). The failure mode in all other specimens was graft pullout.

## 4 Discussion

The present study aimed to compare the biomechanical stability of an allograft fixation system, Shark Screw ACL for graft fixation in ACL reconstruction compared to a conventional interference screw under consideration of slippage onset, local bone density, and intraoperative insertion torque. The data demonstrated that the biomechanical stability during cyclic testing, i.e. slippage, of the Shark Screw ACL was comparable to a standard interference screw. However, the Shark Screw ACL underperformed in ultimate load testing.

To the best knowledge of the authors, this is the first study quantifying the true slippage in different fixation devices by tracking the absolute displacement of the suture firmly attached to the distal end of the ACL graft. Previous investigations have used machine displacement to draw conclusions on fixation stability. Since any ACL graft must be considered as hyperelastic, the slippage of the graft and corresponding stiffness of the fixation are potentially different from the values generated by the displacement of the load frame. A recent study (Benca et al., 2021) has shown that during cyclic loading the machine-measured displacement is mostly a result of graft strain. It was further reported that the machine-measured displacement correlated with graft slippage allowing only for some qualitative evaluation between different fixation devices but stiffness was overall underestimated by up to 400% (Benca et al., 2021). Interestingly, the slippage onset, defined as an initial displacement of at least 0.1 mm, occurs already at relatively low load levels in both implant groups (64.5 and 56.0 N for the interference screw and Shark Screw ACL, respectively). No significant difference could be detected between the two systems in estimated mean survival analysis based on the loads at slippage onset. This result and the fact that the interquartile ranges largely overlap, indicate no inferior slippage performance of the Shark Screw ACL compared to the interference screw. This finding is reinforced by the extent of slippage measured at ultimate load, which was lower in the Shark Screw ACL group (2.5 vs. 2.9 mm) but was not significantly different.

The Shark Screw ACL reached only 63% of the ultimate load and 65% of the estimated mean survival load based on the ultimate load compared to the interference screw. Both findings were significantly different and relevant considering the accompanying confidence intervals. The used interference screw has a significantly higher thread length. The association between implant length and ultimate load is intuitive, however, it could not be confirmed consistently. Testing 28 mm vs. 35 mm tapered screws in sixteen anatomic specimens of tibiae (mean donor age: 38.5 years) revealed significantly higher ultimate loads in the longer screw (595 vs. 825 N, respectively) (Selby et al., 2001). In contrast, a 25 mm and 40 mm interference screw, tested in fourteen tibia specimens (62.6 years) showed similar mean ultimate loads (both 336 N) (Stadelmaier et al., 1999). This discrepancy might be due to different screws, different surgical technique or different donor age, but will remain unclear without further research. Measuring the ultimate load or the load at which the catastrophic failure occurs has been established in previous studies (Sim et al., 2009; Nye et al., 2017; Karkosch et al., 2018) as the standard method for quantifying the primary stability of ACL fixation systems. Ultimate load represents a clearly identifiable and easily reproducible outcome that can be used to compare the findings of different studies and fixation systems. However, the clinical relevance of this variable may be questionable. The graft slippage before reaching the ultimate load must not be large enough to cause a significant degree of laxity and instability of the knee joint. In the present study, the median displacement at ultimate load was below 3.0 mm in both groups. While this appears low, it is important to consider that in some specimens, graft slippage reached clinically significant values (maximum: 7.2 mm in the interference screw group and 11.3 mm in the Shark Screw ACL group) and that in clinical reality, an additional slippage at the femoral side would have to be expected at high loads. Therefore, the ultimate load alone may not be a sufficient indicator for clinical failure (Benca et al., 2021). Thus, a better measure is required to evaluate fixation stability. Load at slippage onset would be of a much higher clinical significance, however, remains challenging to assess.

The ultimate load of the interference screw has been evaluated in numerous studies. (Milano et al., 2006; Milano et al., 2007). The authors have exposed 10 ACL grafts in porcine femora to 1.000 cycles of force-controlled dynamic loading (10–150 N) followed by a load to failure test. The ultimate load reached 678 ± 39 N. Efe et al. (Efe et al., 2010) conducted force-controlled cyclic loading (1.000 cycles, 40–400 N) followed by load to failure tests and additional isolated load to failure tests using 10 porcine tibiae per group to report ultimate loads of 1018 ± 218 N and 864 ± 204 N, respectively. In the present study, the mean ultimate load of the interference screw fixation was considerably lower (277 ± 64 N). The cause of this discrepancy could be the testing of animal and human specimens. Magen et al. (Magen et al., 1999) reported the yield load of an interference screw (standard interference screw, Smith & Nephew DonJoy, Carlsbad, California) to be 350 ± 134 N and 776 ± 155 N in young human (35 years) and porcine tibiae, respectively, with the latter being 122% greater. Similarly, Bailey et al. (Bailey et al., 2004) reported the yield load of an interference screw (9 × 25 mm Standard Interference Screw, Smith & Nephew DonJoy, Carlsbad, CA, United States) to be 87% greater in porcine tibiae than that in young human tibiae. Both studies concluded that the structural properties of a fixation method may not be the same in animal and human tissue. Considering the high donor age in the present cohort, these findings are in line with our results and underline the importance of using human specimens for ACL fixation stability testing.

There have been attempts to estimate *in vivo* loads and strains in the ACL. Li et al. reported the highest *in situ* force in the ACL to be 45 N at 15° knee flexion after applying 200 N quadriceps and 80 N antagonistic hamstrings load (Li et al., 1999) and over 130 N in the ACL during 15° and 30° knee flexion while applying 130 N anterior force to the tibia (Li et al., 2006). In another *in vitro* study (Markolf et al., 2014) a 500 N load was applied axially on the tibia and the knee was exposed to flexion-extension cycles. The mean forces reached the highest values of around 125 N at 25° and 30° degrees during knee extension. Toutoungi et al. (Toutoungi et al., 2000) calculated, based on *in vivo* measurement of limb kinematics and external forces during different rehabilitation exercises, that peak anterior cruciate ligament forces, occurring at knee angles of 35–40°, may reach 0.55× body-weight, i.e., less than 400 N. However, those data have not been validated experimentally. Given the data from the literature, the high specimen age, and the loading in the mechanically worst-case scenario, both fixation types would allow for sufficient fixation under physiological loading conditions. Nevertheless, the authors suggest considering the lower ultimate load levels in clinical routine when using the Shark Screw ACL in the early postsurgical rehabilitation phase, especially for reinjury prevention. Similarly, further biomechanical and clinical studies are required to properly address the intra operative mechanical failure of the allograft implant, as observed in one specimen in the present study.

The significant correlation between the insertion torque and the load at slippage onset and the ultimate load in the interference screw group indicate the possibility of a quick and simple intraoperative estimation of fixation stability. Positive correlations between these parameters were observed in the Shark Screw ACL group as well, however, without reaching significance. One potential explanation for this is that the correlation analyses in this group were based on a smaller number of measurements. Hence, further studies are necessary to shed light on the effect of the insertion torque on implant stability. Insertion torque is expected to be closely related to the local cortical and cancellous bone quantity and quality at the graft-implant interface. Hence, this measure might be more suitable to draw conclusions about implant stability compared to global BMD, although it is available only intra operatively.

This study was performed on a large number of paired human specimens under consideration of true slippage, BMD and insertion torque. Nevertheless, it has several limitations. First, the quality of cancellous bone in younger, more active patients, who normally undergo an ACL reconstruction surgery, is more robust than of that in fresh-frozen anatomic bone specimens from older donors available for *in vitro* studies. This difference suggests much greater fixation strength in clinical routine. It is important to mention that some follow-up studies reported that the clinical outcomes in younger and older patients (older than 40 years) are the same and questioned to establish the age of 40 years as a barrier to successful ACL reconstruction (Barber et al., 1996; Barber et al., 2010). For the same reason, *in vitro* studies should also not be limited to specimens of young donors. While it is difficult to obtain anatomic specimens from younger donors, the authors took this limitation into account by limiting the maximum age to 80 years and by considering the specimen-specific BMD in data analysis. However, the BMD did not correlate with the biomechanical outcomes. Also, due to limited availability of anatomic specimens, sex distribution in the present study – in favor of the male sex – does not fully reflect the injury incidence which is higher in female athletes when practicing the same sport (Anderson et al., 2016). Second, the unidirectional cyclic loading with increasing amplitude applied here does not replicate *in vivo* loading, which remains unknown. The testing methodology limits the application of the findings in routine clinical practice. Simulation of accurate *in vivo* conditions in biomechanical studies is difficult and deviates from simplified models with simulated pathologies. Third, the small sample size resulted in a large deviation of data, however with some detected significance between the groups. Overall, it can be concluded that the Shark Screw ACL allows for sufficient initial fixation stability. Finally, the presented model describes the primary stability of the fixation immediately after surgery and does not consider any biological effects occurring during the weeks and months after surgery. This is important to consider since ACL re-ruptures occur 3.5 (Kyritsis et al., 2016) to 53 (Bourke et al., 2012) months after surgery and the stability of fixation using the Shark Screw ACL would potentially increase due to implant vascularization and bone ingrowth. Therefore, the authors suggest conducting prospective clinical studies in order to quantify the fixation survival, especially in the Shark Screw ACL group.

## 5 Conclusion and outlook

In conclusion, the Shark Screw ACL used for implant fixation in ACL reconstruction demonstrates similar primary stability in terms of graft slippage compared to the interference screw but remains inferior in terms of ultimate load. However, the ultimate load may not be considered as a direct indicator of clinical failure. The clinical relevance of these findings, as well as the anticipated advantage of the Shark Screw ACL for achieving secondary stability, should be further investigated in clinical studies.

## Data Availability

The raw data supporting the conclusion of this article will be made available by the authors upon request due to ethical and legal reasons.

## References

[B1] Anderson M. J. Browning W. M. Urband C. E. Kluczynski M. A. Bisson L. J. (2016). A systematic summary of systematic reviews on the topic of the anterior cruciate ligament. Orthop. J. Of Sports Med. 4, 2325967116634074. 10.1177/2325967116634074 27047983 PMC4794976

[B2] Bailey S. B. Grover D. M. Howell S. M. Hull M. L. (2004). Foam-reinforced elderly human tibia approximates young human tibia better than porcine tibia:A study of the structural properties of three soft tissue fixation devices. Am. J. Of Sports Med. 32, 755–764. 10.1177/0363546503261716 15090394

[B3] Barber F. A. Aziz-Jacobo J. Oro F. B. (2010). Anterior cruciate ligament reconstruction using patellar tendon allograft: An age-dependent outcome evaluation. Arthrosc. J. Arthrosc. Relat. Surg. 26, 488–493. 10.1016/j.arthro.2009.08.022 20362827

[B4] Barber F. A. Elrod B. F. Mcguire D. A. Paulos L. E. (1996). Is an anterior cruciate ligament reconstruction outcome age dependent? Arthrosc. J. Arthrosc. Relat. Surg. 12, 720–725. 10.1016/s0749-8063(96)90177-2 9115562

[B5] Benca E. Zderic I. Caspar J. Knegsel K. V. Hirtler L. Gueorguiev B. (2021). On measuring implant fixation stability in acl reconstruction. Sensors 21, 6632. 10.3390/s21196632 34640951 PMC8513052

[B6] Bourke H. E. Gordon D. J. Salmon L. J. Waller A. Linklater J. Pinczewski L. A. (2012). The outcome at 15 Years of endoscopic anterior cruciate ligament reconstruction using hamstring tendon autograft for ‘isolated’ anterior cruciate ligament rupture. J. Bone Jt. Surg. Br. volume 94-B, 630–637. 10.1302/0301-620x.94b5.28675 22529082

[B7] Brand J. C. Jr. Pienkowski D. Steenlage E. Hamilton D. Johnson D. L. Caborn D. N. (2000b). Interference screw fixation strength of A quadrupled hamstring tendon graft is directly related to bone mineral density and insertion torque. Am. J. Sports Med. 28, 705–710. 10.1177/03635465000280051501 11032229

[B8] Brand J. Weiler A. Caborn D. N. M. Brown C. H. Johnson D. L. (2000a). Graft fixation in cruciate ligament reconstruction. Am. J. Sports Med. 28, 761–774. 10.1177/03635465000280052501 11032238

[B9] Brcic I. Pastl K. Plank H. Igrec J. Schanda J. E. Pastl E. (2021). Incorporation of an allogenic cortical bone graft following arthrodesis of the first metatarsophalangeal joint in A patient with hallux rigidus. Life 11, 473. 10.3390/life11060473 34073841 PMC8225087

[B10] Conner C. S. Perez B. A. Morris R. P. Buckner J. W. Buford W. L. Jr. Ivey F. M. (2010). Three femoral fixation devices for anterior cruciate ligament reconstruction: Comparison of fixation on the lateral cortex versus the anterior cortex. Arthrosc. J. Arthrosc. Relat. Surg. 26, 796–807. 10.1016/j.arthro.2009.10.015 20511038

[B11] Efe T. Bauer J. Herdrich S. Gotzen L. El-Zayat B. F. Schmitt J. (2010). Comparison between bovine bone and titanium interference screws for implant fixation in acl reconstruction: A biomechanical study. Arch. Orthop. Trauma Surg. 130, 993–999. 10.1007/s00402-010-1052-0 20066430

[B12] Eguchi A. Ochi M. Adachi N. Deie M. Nakamae A. Usman M. A. (2014). Mechanical properties of suspensory fixation devices for anterior cruciate ligament reconstruction: Comparison of the fixed-length loop device versus the adjustable-length loop device. Knee 21, 743–748. 10.1016/j.knee.2014.02.009 24613584

[B13] Ehrensberger M. Hohman D. W. Jr. Duncan K. Howard C. Bisson L. (2013). Biomechanical comparison of femoral fixation devices for anterior cruciate ligament reconstruction using A novel testing method. Clin. Biomech. 28, 193–198. 10.1016/j.clinbiomech.2012.12.007 23294848

[B14] Giurea M. Zorilla P. Amis A. A. Aichroth P. (1999). Comparative pull-out and cyclic-loading strength tests of anchorage of hamstring tendon grafts in anterior cruciate ligament reconstruction. Am. J. Sports Med. 27, 621–625. 10.1177/03635465990270051301 10496580

[B15] Halewood C. Hirschmann M. T. Newman S. Hleihil J. Chaimski G. Amis A. A. (2011). The fixation strength of A novel acl soft-tissue graft fixation device compared with conventional interference screws: A biomechanical study *in vitro* . Knee Surg. Sports Traumatol. Arthrosc. 19, 559–567. 10.1007/s00167-010-1255-5 20838764

[B16] Hertel P. Behrend H. Cierpinski T. Musahl V. Widjaja G. (2005). Acl reconstruction using bone-patellar tendon-bone press-fit fixation: 10-Year clinical results. Knee Surg. Sports Traumatol. Arthrosc. 13, 248–255. 10.1007/s00167-004-0606-5 15690197

[B17] Kamelger F. S. Onder U. Schmoelz W. Tecklenburg K. Arora R. Fink C. (2009). Suspensory fixation of grafts in anterior cruciate ligament reconstruction: A biomechanical comparison of 3 implants. Arthrosc. J. Arthrosc. Relat. Surg. 25, 767–776. 10.1016/j.arthro.2009.01.021 19560641

[B18] Karkosch R. F. Ettinger M. Bachmaier S. Wijdicks, C. A. Smith T. (2018). Adjustable-length loop cortical button versus interference screw fixation in quadriceps tendon anterior cruciate ligament reconstruction – a biomechanical *in vitro* study. Clin. Biomech. 60, 60–65. 10.1016/j.clinbiomech.2018.10.001 30321771

[B19] Kousa P. Järvinen T. L. N. Vihavainen M. Kannus P. Järvinen M. (2003a). The fixation strength of six hamstring tendon graft fixation devices in anterior cruciate ligament reconstruction: Part I: Femoral site. Am. J. Sports Med. 31, 174–181. 10.1177/03635465030310020401 12642249

[B20] Kousa P. Järvinen T. L. N. Vihavainen M. Kannus P. Järvinen M. (2003b). The fixation strength of six hamstring tendon graft fixation devices in anterior cruciate ligament reconstruction: Part Ii: Tibial site. Am. J. Sports Med. 31, 182–188. 10.1177/03635465030310020501 12642250

[B21] Kramer D. E. Kalish L. A. Kocher M. S. Yen Y.-M. Micheli L. J. Heyworth B. E. (2020). Complications of bioabsorbable tibial interference screws after anterior cruciate ligament reconstruction in pediatric and adolescent athletes. Orthop. J. Of Sports Med. 8, 232596712090401. 10.1177/2325967120904010 PMC7045297 32154321

[B22] Kyritsis P. Bahr R. Landreau P. Miladi R. Witvrouw E. (2016). Likelihood of acl graft rupture: Not meeting six clinical discharge criteria before return to sport is associated with A four times greater risk of rupture. Br. J. Sports Med. 50, 946–951. 10.1136/bjsports-2015-095908 27215935

[B23] Lajtai G. Schmiedhuber G. Unger F. Aitzetmüller G. Klein M. Noszian I. (2001). Bone tunnel remodeling at the site of biodegradable interference screws used for anterior cruciate ligament reconstruction: 5-Year follow-up. Arthrosc. J. Of Arthrosc. Relat. Surg. 17, 597–602. 10.1053/jars.2001.21535 11447546

[B24] Li G. Papannagari R. E Defrate L. Doo Yoo J. Eun Park S. J Gill T. (2006). Comparison of the acl and acl graft forces before and after acl reconstruction an *in-vitro* robotic investigation. Acta Orthop. 77, 267–274. 10.1080/17453670610046019 16752289

[B25] Li G. Rudy T. W. Sakane M. Kanamori A. Ma C. B. Woo S. L. Y. (1999). The importance of quadriceps and hamstring muscle loading on knee kinematics and *in-situ* forces in the acl. J. Of Biomechanics 32, 395–400. 10.1016/s0021-9290(98)00181-x 10213029

[B26] Magen H. E. Howell S. M. Hull M. L. (1999). Structural properties of six tibial fixation methods for anterior cruciate ligament soft tissue grafts. Am. J. Sports Med. 27, 35–43. 10.1177/03635465990270011401 9934416

[B27] Malek M. Deluca J. Verch D. Kunkle K. (1996). Arthroscopically assisted acl reconstruction using central third patellar tendon autograft with press fit femoral fixation. Instr. Course Lect. 45, 287–295. 8727748

[B28] Markolf K. L. Jackson S. R. Foster B. Mcallister D. R. (2014). Acl forces and knee kinematics produced by axial tibial compression during A passive flexion–extension cycle. J. Orthop. Res. 32, 89–95. 10.1002/jor.22476 23996893

[B29] Mayr R. Heinrichs C. H. Eichinger M. Coppola C. Schmoelz W. Attal R. (2015). Biomechanical comparison of 2 anterior cruciate ligament graft preparation techniques for tibial fixation:adjustable-length loop cortical button or interference screw. Am. J. Sports Med. 43, 1380–1385. 10.1177/0363546515574062 25767269

[B30] Milano G. Mulas P. D. Ziranu F. Deriu L. Fabbriciani C. (2007). Comparison of femoral fixation methods for anterior cruciate ligament reconstruction with patellar tendon graft: A mechanical analysis in porcine knees. Knee Surg. Sports Traumatol. Arthrosc. 15, 733–738. 10.1007/s00167-006-0269-5 17295042

[B31] Milano G. Mulas P. D. Ziranu F. Piras S. Manunta A. Fabbriciani C. (2006). Comparison between different femoral fixation devices for acl reconstruction with doubled hamstring tendon graft: A biomechanical analysis. Arthrosc. J. Arthrosc. Relat. Surg. 22, 660–668. 10.1016/j.arthro.2006.04.082 16762706

[B32] Miller C. D. Gerdeman A. C. Bennett C. G. Hart J. M. Miller M. D. (2010). A biomechanical comparison of the endobutton Cl using transtibial drilling and endobutton direct using anteromedial arthroscopic drilling. Arthrosc. J. Arthrosc. Relat. Surg. 26, 1311–1317. 10.1016/j.arthro.2010.02.018 20887930

[B33] Monaco E. Labianca L. Speranza A. Agrò A. M. Camillieri G. D’arrigo C. (2010). Biomechanical evaluation of different anterior cruciate ligament fixation techniques for hamstring graft. J. Of Orthop. Sci. 15, 125–131. 10.1007/s00776-009-1417-9 20151262

[B34] Nye D. D. Mitchell W. R. Liu W. Ostrander R. V. (2017). Biomechanical comparison of fixed-loop and adjustable-loop cortical suspensory devices for metaphyseal femoral-sided soft tissue graft fixation in anatomic anterior cruciate ligament reconstruction using A porcine model. Arthrosc. J. Arthrosc. Relat. Surg. 33, 1225–1232.E1. 10.1016/j.arthro.2016.12.014 28216289

[B35] Pastl K. Schimetta W. (2021). The application of an allogeneic bone screw for osteosynthesis in hand and foot surgery: A case series. Archives Of Orthop. And Trauma Surg. 42 (10), 2567–2575. 10.1007/s00402-021-03880-6 PMC9474387 33834287

[B36] Petre B. M. Smith S. D. Jansson K. S. De Meijer P.-P. Hackett T. R. Laprade R. F. (2013). Femoral cortical suspension devices for soft tissue anterior cruciate ligament reconstruction:A comparative biomechanical study. Am. J. Sports Med. 41, 416–422. 10.1177/0363546512469875 23263298

[B37] Pinczewski L. A. Lyman J. Salmon L. J. Russell V. J. Roe J. Linklater J. (2007). A 10-year comparison of anterior cruciate ligament reconstructions with hamstring tendon and patellar tendon autograft:A controlled, prospective trial. Am. J. Sports Med. 35, 564–574. 10.1177/0363546506296042 17261567

[B38] Scannell B. P. Loeffler B. J. Hoenig M. Peindl R. D. D'alessandro D. F. Connor P. M. (2015a). Biomechanical comparison of hamstring tendon fixation devices for anterior cruciate ligament reconstruction: Part 1. Five femoral devices. Am. J. Orthop. 44, 32–36. 25566554

[B39] Scannell B. P. Loeffler B. J. Hoenig M. Peindl R. D. D'alessandro D. F. Connor P. M. (2015b). Biomechanical comparison of hamstring tendon fixation devices for anterior cruciate ligament reconstruction: Part 2. Four tibial devices. Am. J. Orthop. 44, 82–85. 25658077

[B40] Schlichting K. Dahne M. Weiler A. (2006). Biodegradable composite implants. Sports Med. And Arthrosc. Rev. 14, 169–176. 10.1097/00132585-200609000-00009 17135964

[B41] Selby J. B. Johnson D. L. Hester P. Caborn D. N. (2001). Effect of screw length on bioabsorbable interference screw fixation in A tibial bone tunnel. Am. J. Sports Med. 29, 614–619. 10.1177/03635465010290051401 11573920

[B42] Sim J. A. Kwak J. H. Yang S. H. Lee B. K. (2009). Comparative biomechanical study of the ligament Plate® and other fixation devices in acl reconstruction. Int. Orthop. 33, 1269–1274. 10.1007/s00264-008-0653-5 18923833 PMC2899102

[B43] Smith P. A. Piepenbrink M. Smith S. K. Bachmaier S. Bedi A. Wijdicks C. A. (2018). Adjustable- versus fixed-loop devices for femoral fixation in acl reconstruction: An *in vitro* full-construct biomechanical study of surgical technique–based tibial fixation and graft preparation. Orthop. J. Of Sports Med. 6, 232596711876874. 10.1177/2325967118768743 PMC5954336 29780843

[B44] Speirs A. Simon D. Lapner P. (2010). Evaluation of A new femoral fixation device in A simulated anterior cruciate ligament reconstruction. Arthrosc. J. Arthrosc. Relat. Surg. 26, 351–357. 10.1016/j.arthro.2009.08.016 20206045

[B45] Stadelmaier D. M. Lowe W. R. Ilahi O. A. Noble P. C. Kohl H. W. (1999). Cyclic pull-out strength of hamstring tendon graft fixation with soft tissue interference screws. Am. J. Sports Med. 27, 778–783. 10.1177/03635465990270061501 10569365

[B46] Toutoungi D. E. Lu T. W. Leardini A. Catani F. O’connor J. J. (2000). Cruciate ligament forces in the human knee during rehabilitation exercises. Clin. Biomech. 15, 176–187. 10.1016/s0268-0033(99)00063-7 10656979

[B47] Trump M. Palathinkal D. M. Beaupre L. Otto D. Leung P. Amirfazli A. (2011). *In vitro* biomechanical testing of anterior cruciate ligament reconstruction: Traditional versus physiologically relevant load analysis. Knee 18, 193–201. 10.1016/j.knee.2010.04.011 20570155

[B48] Warden W. H. Friedman R. Teresi L. M. Jackson D. W. (1999). Magnetic resonance imaging of bioabsorbable polylactic acid interference screws during the first 2 Years after anterior cruciate ligament reconstruction. Arthrosc. J. Of Arthrosc. Relat. Surg. 15, 474–480. 10.1053/ar.1999.v15.015047 10424550

